# Personalized Oncology Suite: integrating next-generation sequencing data and whole-slide bioimages

**DOI:** 10.1186/1471-2105-15-306

**Published:** 2014-09-18

**Authors:** Andreas Dander, Matthias Baldauf, Michael Sperk, Stephan Pabinger, Benjamin Hiltpolt, Zlatko Trajanoski

**Affiliations:** Division for Bioinformatics, Biocenter, Innsbruck Medical University, Innrain 80-82, 6020 Innsbruck, Austria; Oncotyrol GmbH, Center for Personalized Cancer Medicine, Karl-Kapferer-Strasse 5, 6020 Innsbruck, Austria; AIT-Austrian Institute of Technology, Health & Environment Department, Molecular Diagnostics, Muthgasse 11, 1190 Vienna, Austria

**Keywords:** Personalized oncology, Data integration, Next-generation sequencing, Whole-slide bioimaging, Application, Open-source

## Abstract

**Background:**

Cancer immunotherapy has recently entered a remarkable renaissance phase with the approval of several agents for treatment. Cancer treatment platforms have demonstrated profound tumor regressions including complete cure in patients with metastatic cancer. Moreover, technological advances in next-generation sequencing (NGS) as well as the development of devices for scanning whole-slide bioimages from tissue sections and image analysis software for quantitation of tumor-infiltrating lymphocytes (TILs) allow, for the first time, the development of personalized cancer immunotherapies that target patient specific mutations. However, there is currently no bioinformatics solution that supports the integration of these heterogeneous datasets.

**Results:**

We have developed a bioinformatics platform – Personalized Oncology Suite (POS) – that integrates clinical data, NGS data and whole-slide bioimages from tissue sections. POS is a web-based platform that is scalable, flexible and expandable. The underlying database is based on a data warehouse schema, which is used to integrate information from different sources. POS stores clinical data, genomic data (SNPs and INDELs identified from NGS analysis), and scanned whole-slide images. It features a genome browser as well as access to several instances of the bioimage management application Bisque. POS provides different visualization techniques and offers sophisticated upload and download possibilities. The modular architecture of POS allows the community to easily modify and extend the application.

**Conclusions:**

The web-based integration of clinical, NGS, and imaging data represents a valuable resource for clinical researchers and future application in medical oncology. POS can be used not only in the context of cancer immunology but also in other studies in which NGS data and images of tissue sections are generated. The application is open-source and can be downloaded at http://www.icbi.at/POS.

**Electronic supplementary material:**

The online version of this article (doi:10.1186/1471-2105-15-306) contains supplementary material, which is available to authorized users.

## Background

Cancer immunotherapy has recently entered a remarkable renaissance phase with the approval of several agents for treatment [1]. Cancer immunotherapies that involve the use of the adaptive immune system, such as anti-checkpoint antibodies and adoptive T-cell therapies, have demonstrated profound tumor regressions including complete cure in patients with metastatic cancer. Technological advances in next-generation sequencing (NGS) as well as the development of devices for scanning whole-slide images from tissue sections and image analysis software for quantitation of tumor-infiltrating lymphocytes (TILs) allow, for the first time, the development of personalized cancer immunotherapies that target patient specific mutations. The use of NGS technologies to characterize tumor samples enables one not only to comprehensively study the interactions between human cancers and the immune system, but also to identify targets for patient stratification. Moreover, the quantitation of TILs will improve therapeutic efficacy, even in the absence of immunotherapy. It will enable a precise characterization of the immune infiltrates in the tumor and will help to identify mechanisms of tumor regression and disentangle the complex tumor-immune cell interactions. For example, understanding the molecular basis of the interactions between cytotoxic chemotherapeutics or targeted anti-cancer agents and the immune system is essential for the development of optimal therapeutic schemes and in the long run will result in clinical benefit for the patients.

However, the real value of the disparate datasets can be truly exploited only when the data are integrated. In our experience it is of utmost importance to establish a local database hosting only the necessary data. Only pre-processed and normalized data will be stored in a dedicated relational database whereas primary data are archived at separate locations including public repositories [2]. To this end, a database that integrates clinical, NGS, and bioimaging data would be extremely helpful for clinical cancer research and in near future also for routine applications in medical oncology. However, to the best of our knowledge there is currently no application that supports this integration. As of today there are different applications integrating either clinical data and NGS data or bioimages (Table 1) but no integrated solution has been created. We therefore developed the bioinformatics platform Personalized Oncology Suite (POS) to overcome this bottleneck and support the researchers working in this exciting field.

**Table 1:** **This table compares POS with other applications in the context of data integration**

Application	Edit	Clinical	TNM	Sequencing	Vis.	Bioimages	Public	Ref.
BioIMAX	☑	☒	☒	☒	☒	Whole-slide	☒	[3]
Bisque	☑	☒	☒	☒	☒	Whole-slide	☒	[4]
Galaxy LIMS	☑	☒	☒	☑	☒	☒	☒	[5]
NG6	☒	☒	☒	Raw 454 and HiSeq	☒	☒	☒	[6]
NGS tools	☑	☒	☒	Raw Illumina	☒	☒	DAS [7]	[8]
OMERO	☑	☒	☒	☒	☒	☒	☒	[9]
ONCO-i2b2	☒	☑	☑	☒	☒	☒	SNOMED	[10]
openBIS	☑	☒	☒	Raw Illumina	☒	☒	☒	[11]
Taverna	☒	☒	☒	☑	☒	☒	☑	[12]
**POS**	☑	☑	☑	vcf Files	☑	Whole-slide	COSMIC [13]	-

The columns *Clinical* and *TNM* show if these data types are available. *Sequencing* depicts which type of next-generation sequencing data can be uploaded, and the column *Vis*. shows if mutations can be visualized. The column *Bioimages* shows which type of images can be used and the final column *Public* states available public annotations.

## Implementation

In order to be scalable, flexible, and expandable, POS makes use of state of the art software engineering techniques and architectures like the Java Enterprise Edition 6 (J2EE 6) technology stack. It is a web-based platform relying on the JBoss Application Server in version 7.1.1. The modular three-tier architecture (web frontend, application core, database backend) and the release under the open-source license GNU AGPL enables the community to easily modify and extend the application with further functionalities.

**Figure 1:**
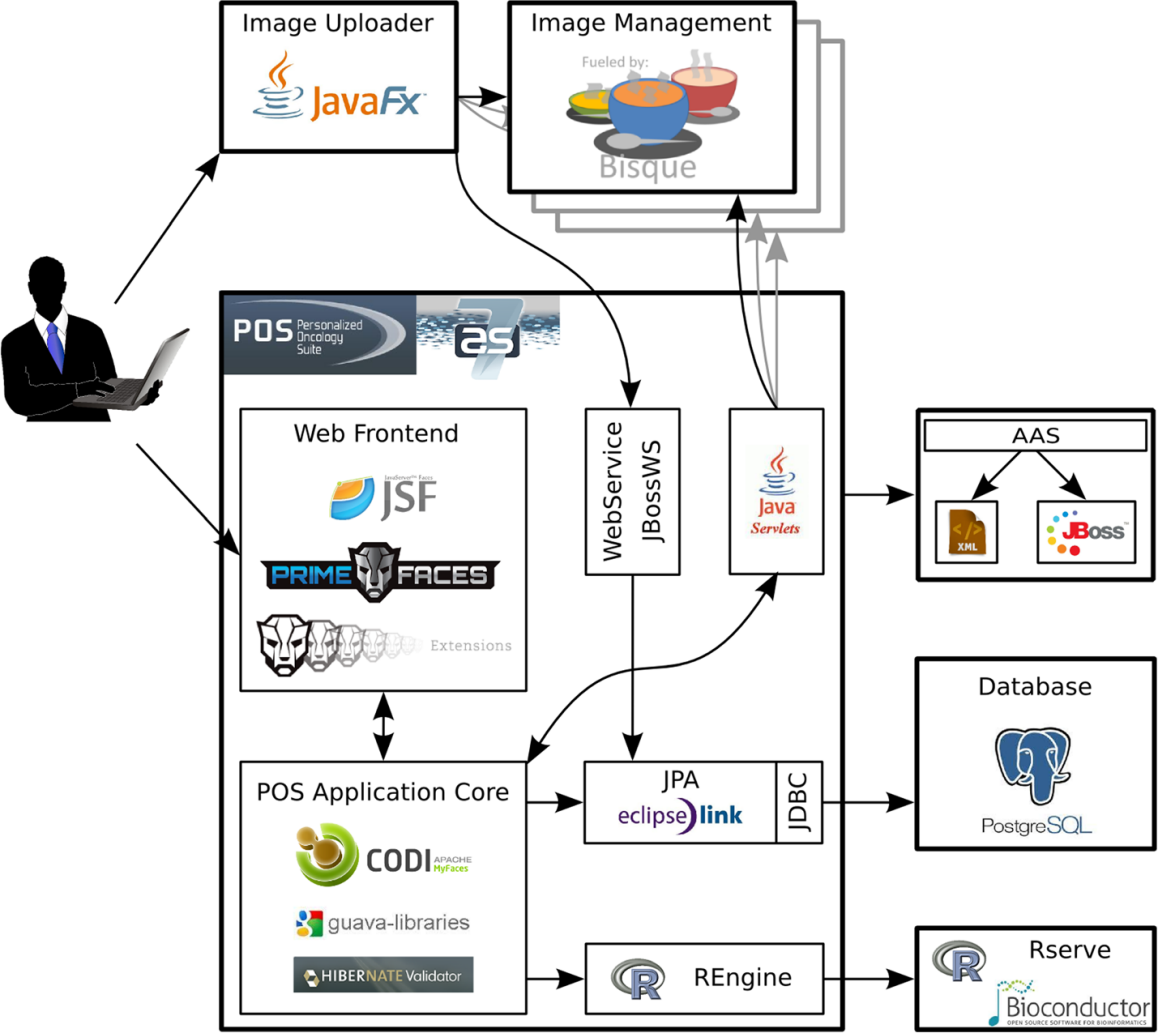
**Software architecture of POS.** The JBoss Application Server of POS is shown as the central rectangle containing different JSF libraries. On the right hand side the attached Authorization and Authentication System (AAS) is depicted. POS uses PostgreSQL as database management system and applies EclipseLink for the object-relational mapping. The Bioconductor package Gviz handles rendering of the genome browser tracks, and the R package Rserver provides an R server available through a network connection. On top, distributed Bisque instances are shown. The POS Image Uploader, a JavaFX based standalone application, is outlined on the top left in the figure. This application enables users to upload several images at once.

Figure 1 outlines the software architecture of POS and depicts the main used libraries. PrimeFaces and PrimeFaces Extensions are used for the creation of JSF components, whereas Hibernate Validator provides input validation of user entries. As access scopes of Java Beans are crucial within JavaEE applications, Apache CODI is used to include additional scopes. Due to the fact that POS deals with different types of collections, the Guava libraries were chosen to support POS with a set of helpful functionalities regarding collections. Used Bisque instances are shown at the top of Figure 1. Furthermore, the standalone application POS Image Uploader allows batch uploading of numerous images at once.

The underlying database is based on a data warehouse schema. This schema was chosen as it is widely used for integrating information from different sources. All defined entities are outlined in Additional file 1. POS uses the Java library EclipseLink for object-relational mapping. In the default configuration POS runs with PostgreSQL, but can be easily exchanged with another relational database.

## Results and discussion

The Personalized Oncology Suite is an application combining biological and clinical data into one integrated solution. In this context, clinical data comprises information about cancer patients, TNM staging, and density values of TILs used for immune score estimation. Biological data describes mutations found via next-generation sequencing and whole-slide bioimages, which can be uploaded to the application. POS features different types of visualization techniques for all integrated data types. Furthermore, publicly available data from the COSMIC database [13] is integrated into POS. As data types are stored in different file formats, POS includes several data import and export possibilities for the most important formats. In addition, different filters on the data can be applied either individually or in combination (e.g. age at diagnosis and TNM stage). In the current implementation queries can be done only for single modalities (e.g. genetic features, images, or clinical parameters). Furthermore, patients can be selected based on their UICC stage or Immunoscore by using a range slider. POS features different user interface languages using internationalization and is fault tolerant as all inputs are validated. Furthermore, POS implements exception handling as well as an intelligent logging functionality.

**Figure 2:**
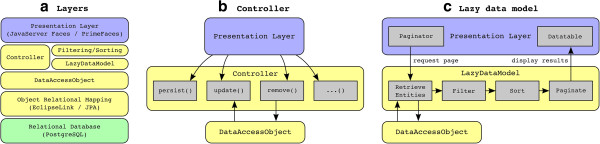
**Software layers of POS. a)** The JSF based presentation layer on top (blue) access the relational database (green) via the Java backend. This backend can be accessed via the classes Controller or LazyDataModel. These classes make use of the DataAccessObject which provides a single access point to the relational database by using object-relational mapping. **b)** The Controller is responsible for secure CRUD (create, read, update and delete) operations. **c)** The LazyDataModel is used for providing immutable, filtered, sorted, and paginated collections to the presentation layer.

POS has been released under the open-source license GNU AGPL to allow integration of new components from the scientific community. The source code can be accessed via the project website http://www.icbi.at/POS. Figure 2 outlines the different layers between the JSF based frontend and the relational database.

### Clinical data and tumor staging

POS integrates clinical data of cancer patients including various attributes such as gender, date of diagnosis, disease duration, adjuvant therapy, and relapse. We have included compliant measures in the design of the software ensuring that patient-identifying data such as name, academic title, address, telephone number, e-mail address, date and place of birth, as well as date and place of death [14] are not collected. Patients are uniquely defined by alphanumerical identifiers. Thus, data from multiple clinical visits or any other information can be unambiguously assigned to an individual patient.

### Tumor staging

In addition to clinical data, POS integrates the TNM cancer staging system. The T, N, and M categories are stored separately within the database and the resulting AJCC/UICC stage (0-IV) is determined from this information. POS facilitates the creation of descriptive plots for comparing patients in different TNM stages. These plots can also be used for comparison of data among different participating institutes, provided the user has permission to access the information. In addition to input data manually, clinical and staging data can be uploaded to POS in CSV format. Furthermore, these data types can be exported as XLS, CSV and PDF files.

### Next-generation sequencing data

With the use of next-generation sequencing, biologists are able to determine the order of nucleotide bases composing the DNA. The identification of somatic mutations can be performed by sequencing tumor/normal pairs and subsequently comparing cancerous to healthy tissue. Several different applications exist which are able to analyze tumor/normal pairs regarding their somatic SNPs and INDELs [15]. Since there are a plethora of methods for analyses of NGS data, the design of the software focused on the integration of disparate data types rather than the analyses of raw data. Furthermore, as the methods and available tools improve at fast pace, integration of processed data makes the system more flexible and versatile. POS is able to integrate somatic SNPs and INDELs identified by such experiments. For uploading this type of data, users first need to define an analysis, which can be used to attach called mutations. POS supports data upload via VCF (Variant Call Format) files or by manual input of mutations.

### Genome browser

**Figure 3:**
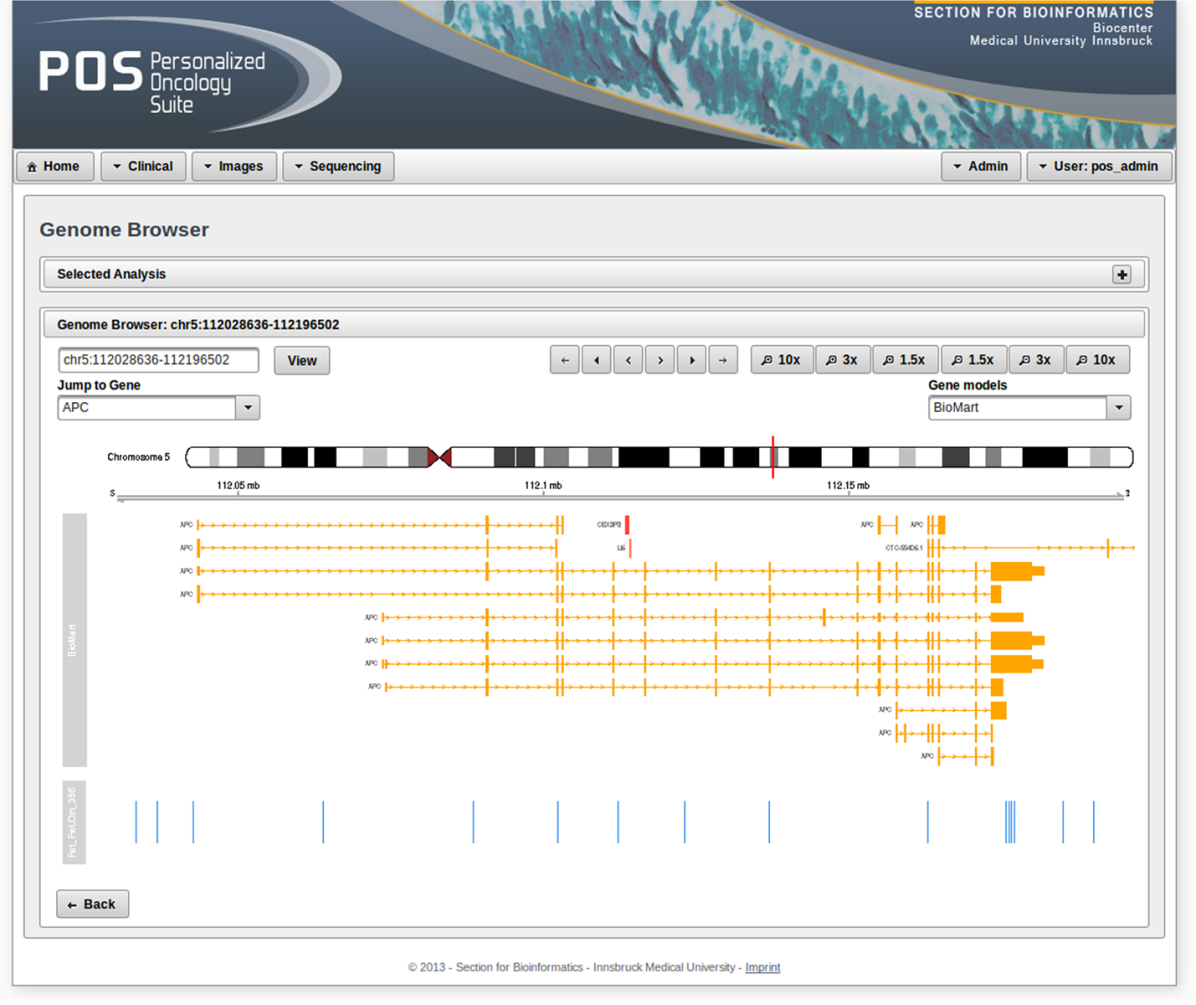
**View of the genome browser.** A genome browser visualizing mutations in the context of the reference genome including publicly available annotations is shown. The first track depicts the ideogram of the chosen chromosome, followed by an axis showing its genomic coordinates. Next, publicly available annotations derived from BioMart [16] are depicted. The bottom track holds information about uploaded mutations. The patient name and the shown mutations were randomly generated.

As visualization of identified mutations tremendously supports the interpretation of these results, genome browsers have been developed to display mutations in the context of a reference genome [15]. POS includes a genome browser that features a combined view of different tracks, each containing a dedicated plot. Figure 3 shows a screenshot of the genome browser view within POS. On top of the genome browser panel, the region of interest can be specified by defining chromosome number, start and end position or by choosing a gene of interest. The user can select several patients at once and mutations for each patient will be displayed in separate tracks.

### Bioimages

Whole-slide bioimages can be used to estimate the density of TILs. However, due to the size of the files the required disk space can be very large. Therefore, the raw image files are not stored in POS. In order to integrate whole-slide images within POS, the application connects to several instances of the bioimage management application Bisque [4] as outlined in Figure 1. POS supports the following formats: 1) SVS format from Aperio, 2) VSI format from Olympus, 3) Hamamatsu formats NDPI and VMS, 4) TIFF format from Trestle, 5) Leica’s SCN format, 6) formats ZVI and CZI from Zeiss, and 7) OME.TIFF format from the Open Microscopy Environment OME [17, 18].

### Image upload

POS supports different ways for uploading images. The first option is a direct upload module, where a patient needs to be selected before the upload starts. The second possibility allows access to already uploaded images within a connected Bisque instance and their assignment to selected patients in POS. The third option is an external upload application supporting the batch upload of multiple images at once. This application is also able to assign the correct relations between uploaded images and patients within POS.

### Bisque connection

**Figure 4:**
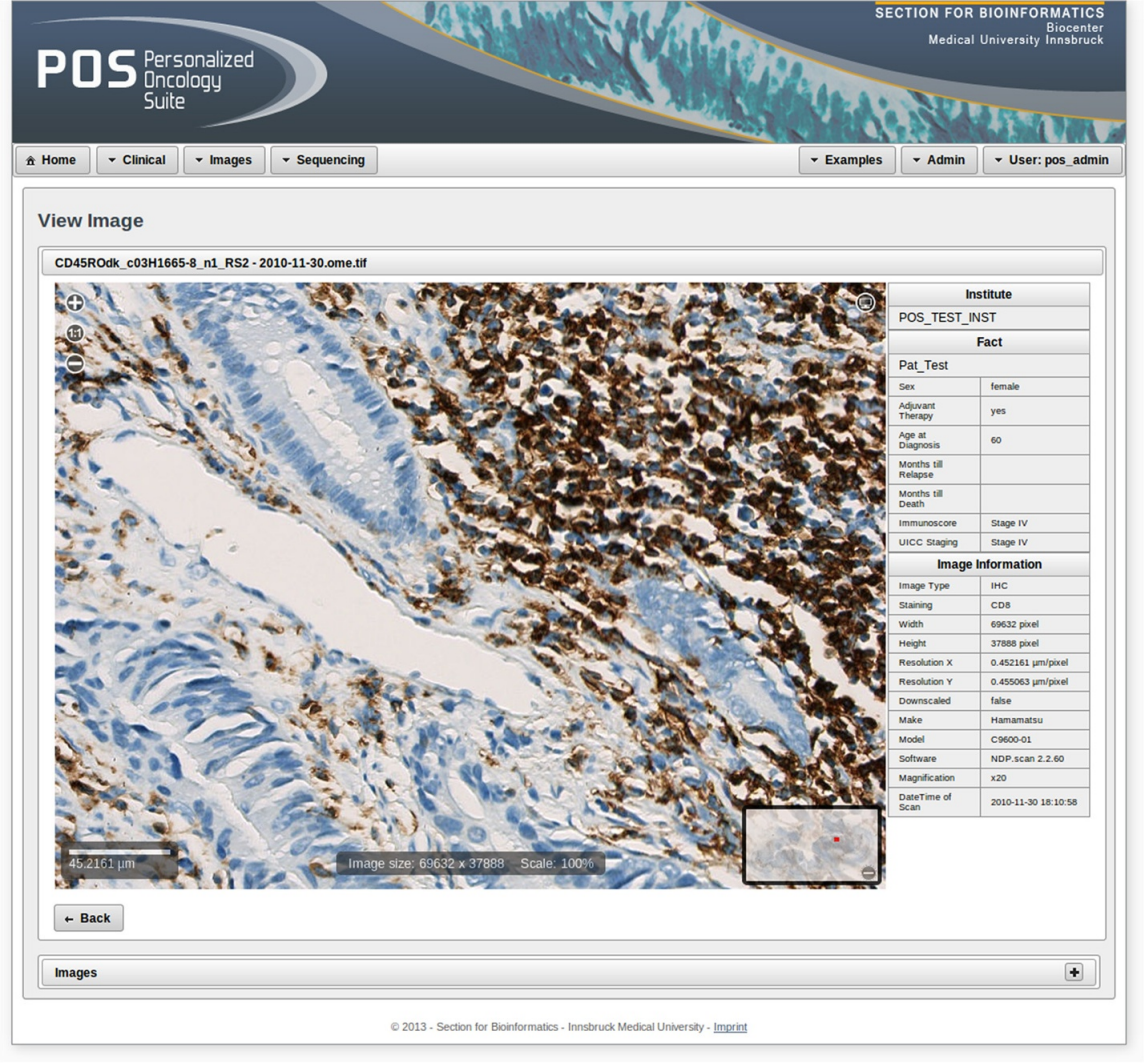
**Image viewer.** This Figure depicts a screenshot of an example of a whole-slide bioimage. The image is stored at an external Bisque instance and only tiles that correspond to the current image section are transferred to POS. General information about the patient and the image are depicted on the right hand side. Various attributes like width, height, resolution, and information about the scanner are directly fetched from Bisque. Shown clinical data were randomly generated.

The connection to Bisque instances is administered by POS, which handles correct URL, port, username and password settings. In addition, tailored Java servlets for managing the encrypted communication between POS and Bisque have been developed. Downscaling and tiling of the images is performed by the connected Bisque systems. POS is able to display the created tiles in a Google maps like manner, using an adapted version of the interactive JavaScript widget PanoJS3. Hence, only tiles of the currently displayed region are fetched from Bisque. A screenshot of the POS image viewer is displayed in Figure 4.

### Flexible batch upload module

As the JSF based image upload form of POS does not support batch upload of several images at once, the standalone application POS Image Uploader has been developed. This application is based on JavaFX and can be started on all systems where Java is installed. Figure 1 depicts this standalone application at the top. The POS Image Uploader is able to acquire the URL and corresponding login information of Bisque instances directly from POS. During the batch upload, each image is uploaded to a selected Bisque instance. Bisque returns identifiers for each uploaded image, which are stored within the POS database and linked to related patients. The communication between the standalone tool and the web-based application is managed by tailored web services.

### Authorization and authentication system

As POS holds confidential and patient related data, the application needs to be secured. POS is secured by an authorization and authentication system (AAS) where participating users and institutes can be configured within this easy to use user management system [19]. The backend of the used AAS can either be a simple XML file or a web-based application running on a dedicated JBoss Application Server (see Figure 1). For tailored user access, POS applies different user roles, provided by the AAS. Access roles are defined for clinical, NGS, and imaging data as well as for administration. In order to provide custom access profiles, users can be assigned to several roles at once. Furthermore, all web services, used by the POS Image Uploader, authenticate each single connection via the AAS.

Finally, POS users can also share their data with other participating users, with the possibility to restrict access to certain data types. It should be noted that as access to patient related data needs to be secured, the implemented sharing concept and the AAS do not support public access to any data type. Rather, users can specify the type of data that can be shared with other users including images, genetic data, or clinical data. If an external user wants access to this integrated data a request must be performed and all participating institutes will be informed about this request.

### Installation

As POS is a web-based application, it can be accessed from any operating system. The only requirements are an up to date web browser and a network connection to the POS server. The server itself is able to run on any operating system with installed Java and R, and has been extensively tested on Linux and Windows machines. The database backend is interchangeable - POS has been tested with the database systems PostgreSQL and MySQL. A detailed installation guide for POS is available at the project home page, accessible at http://www.icbi.at/POS. As POS makes use of several Bisque installations, an installation guide for Bisque is provided at the same location.

## Conclusions

POS is a web-based application combining clinical and biomolecular data including NGS data and bioimages. Due to its modular and flexible architecture it can be easily extended and adapted to different requirements. POS provides an intuitive user interface supporting data upload, manipulation and visualization of integrated data types. In summary, POS represents an effective solution for current challenges in clinical cancer research. The suite can be used not only in the context of personalized cancer immunotherapy but also in other studies where NGS data and images of tissue sections are generated.

## Availability and requirements

**Project name:** Personalized Oncology Suite (POS)

**Project home page:**http://www.icbi.at/POS

**Operating system:** Platform independent

**Programming language:** Java, R/Bioconductor

**License:** GNU AGPL

**Any restrictions to use by non-academics:** no

## Electronic supplementary material

Additional file 1: **Database schema of the Personalized Oncology Suite.** The database schema of POS is based on a data warehouse schema. Therefore, the central entity represents patients. Each *patient* belongs to an *institute*, which stores the *externalid* referencing an institute within the attached Authorization and Authentication System. It can be seen that each institute can be connected to an *imagerepository* containing information about the connection to a Bisque instance. The entity *institutesharing* manages information about shared data. *Clinicaldata* as well as *tnm* and *immunoscore* for staging of cancer are related to the entity *patient*. For the integration of somatic mutations within POS the entities *snp* and *indel* are used. The entity *analysis* contains metadata about the next-generation sequencing itself. *Image* manages the attributes *externalimageid* and *externalresourceid* which are IDs used for accessing the image within Bisque. The attached *imagetype* contains information about the staining of the image. All entities with a name like <*name*>_*audittrail* hold information about documented changes made to the attached entity <*name*>. It is shown that the *timestamp*, the name of the *author* and the performed *changes* are recorded within these entities. Several entities comprise a *deleted* flag. If such an entity gets deleted it will not be removed within the database, but will not be shown in the frontend. This has the advantage that deleted entities can be restored by a database administrator. (PNG 1 MB)

## Abbreviations

AAS: Authorization and Authentication System
AGPL: Affero General Public License
COSMIC: Catalogue of Somatic Mutations in Cancer
CRUD: Create, Read, Update and Delete
INDEL: Small insertion or deletion
JSF: JavaServer Faces
POS: Personalized Oncology Suite
SNP: Single-nucleotide polymorphism
TIL: Tumor-infiltrating lymphocyte
TNM: Classification of Malignant Tumors
URL: Uniform Resource Locator
VCF: Variant Call Format.
