# Supplementary figures and images for: Personalized Oncology Suite: integrating next-generation sequencing data and whole-slide bioimages
Source: BMC Bioinformatics. 2014 Sep 18;15(1):306. doi: 10.1186/1471-2105-15-306 (PMC4261581; doi:10.1186/1471-2105-15-306)

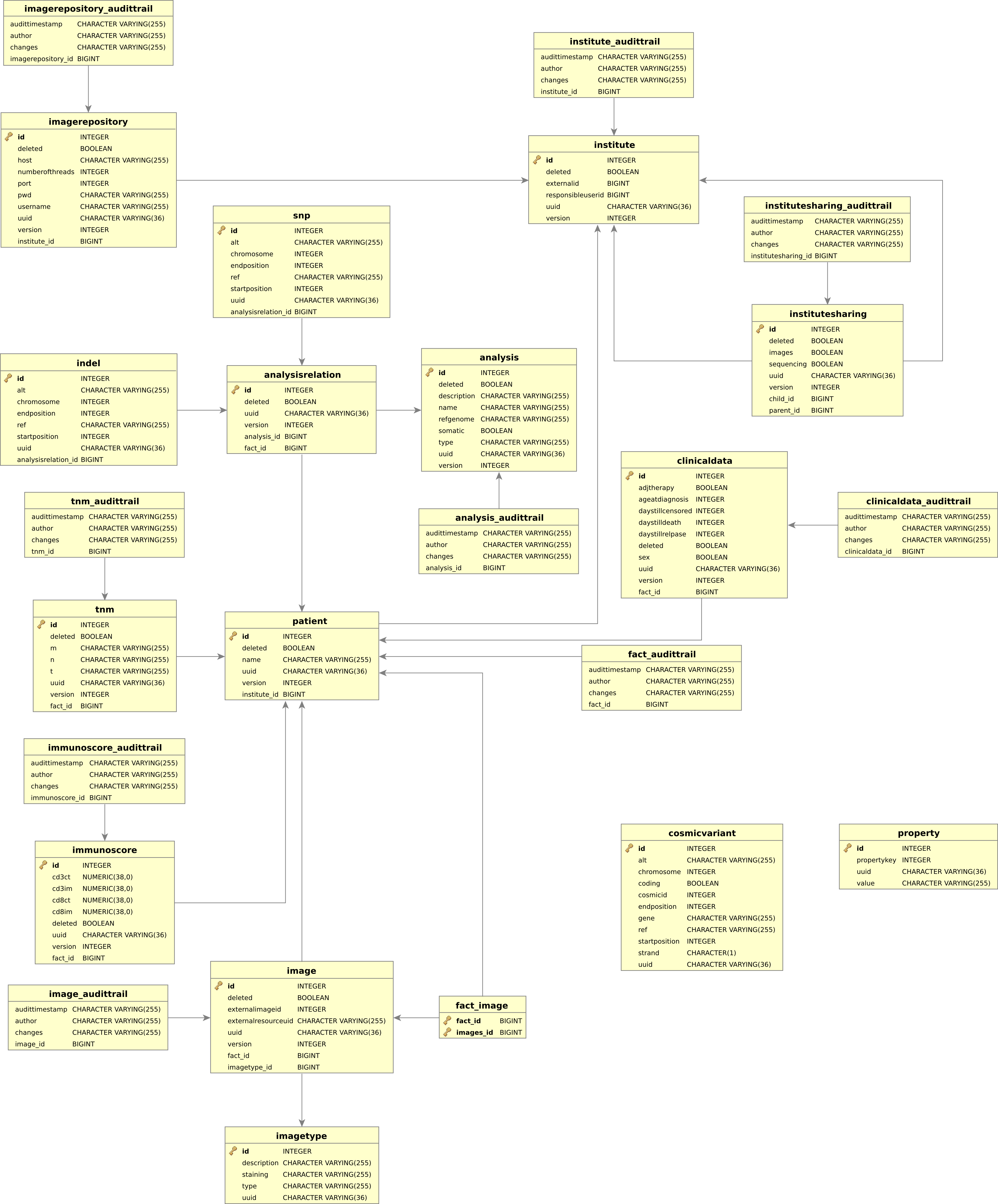

Supplement: Supplementary file 1 — Additional file 1: Database schema of the Personalized Oncology Suite. The database schema of POS is based on a data warehouse schema. Therefore, the central entity represents patients. Each patient belongs to an institute, which stores the externalid referencing an institute within the attached Authorization and Authentication System. It can be seen that each institute can be connected to an imagerepository containing information about the connection to a Bisque instance. The entity institutesharing manages information about shared data. Clinicaldata as well as tnm and immunoscore for staging of cancer are related to the entity patient. For the integration of somatic mutations within POS the entities snp and indel are used. The entity analysis contains metadata about the next-generation sequencing itself. Image manages the attributes externalimageid and externalresourceid which are IDs used for accessing the image within Bisque. The attached imagetype contains information about the staining of the image. All entities with a name like _audittrail hold information about documented changes made to the attached entity . It is shown that the timestamp, the name of the author and the performed changes are recorded within these entities. Several entities comprise a deleted flag. If such an entity gets deleted it will not be removed within the database, but will not be shown in the frontend. This has the advantage that deleted entities can be restored by a database administrator. (PNG 1 MB) [file 12859_2014_6629_MOESM1_ESM.png]
